# Supplementary material for: Characterization and Localization of Sol g 2.1 Protein from Solenopsis geminata Fire Ant Venom in the Central Nervous System of Injected Crickets (Acheta domestica)
Source: Int J Mol Sci. 2023 Oct 1;24(19):14814. doi: 10.3390/ijms241914814 (PMC10573061; doi:10.3390/ijms241914814)
Supplement: Supplementary file 1 [file ijms-24-14814-s001.zip › ijms-2618190-supplementary-final.pdf]

Supplement figures

```
TATTTTCAGGGCGCCATGGGATCCGGAATTCAACATAATGAAGAACTAAAAGTTATACAT    27
 Y  F  Q  G  A  M  G  S  G  I  Q  H  N  E  E  L  K  V  I  H     9

AAGGATATAGCAAAATGTGCAAGAACATTACCAAAATGCGTAAATCAACCAGATGATCCG    87
 K  D  I  A  K  C  A  R  T  L  P  K  C  V  N  Q  P  D  D  P     29

TTAGCTAGAGTCGATGTATGGCATTGTGCTATGGCCAAGCGTGGCGTATATGACAACCCA    147
 L  A  R  V  D  V  W  H  C  A  M  A  K  R  G  V  Y  D  N  P     49

GCTCCAGCTGTTATAAAAGAAAAAAATTTTAAAGTATGCTCCAAGATTATCACTGATCCC    207
 A  P  A  V  I  K  E  K  N  F  K  V  C  S  K  I  I  T  D  P     69

GCTAATGTCGAAAATTGCAAGAAAGTTATTTCTAGATGTGTAGATAGAGAGACTCAACGC    267
 A  N  V  E  N  C  K  K  V  I  S  R  C  V  D  R  E  T  Q  R     89

CCAAGATCCAACAGACAGAAAGCAATAAATATAACAGGATGTATTTTAAGAGCTGGTGTG    327
 P  R  S  N  R  Q  K  A  I  N  I  T  G  C  I  L  R  A  G  V     109

GTGGAGACTACAGTGCTAGCCCGTAAAAAATGA                               360
V  E  T  T  V  L  A  R  K  K  *                                 119
```

Figure S1. Shows DNA sequence and deduced amino acid encoding of Sol g 2.1 protein. Blue box indicates the first amino acid of the mature protein, which was determined by Edman degradation sequencing. (*) represents stop codon.

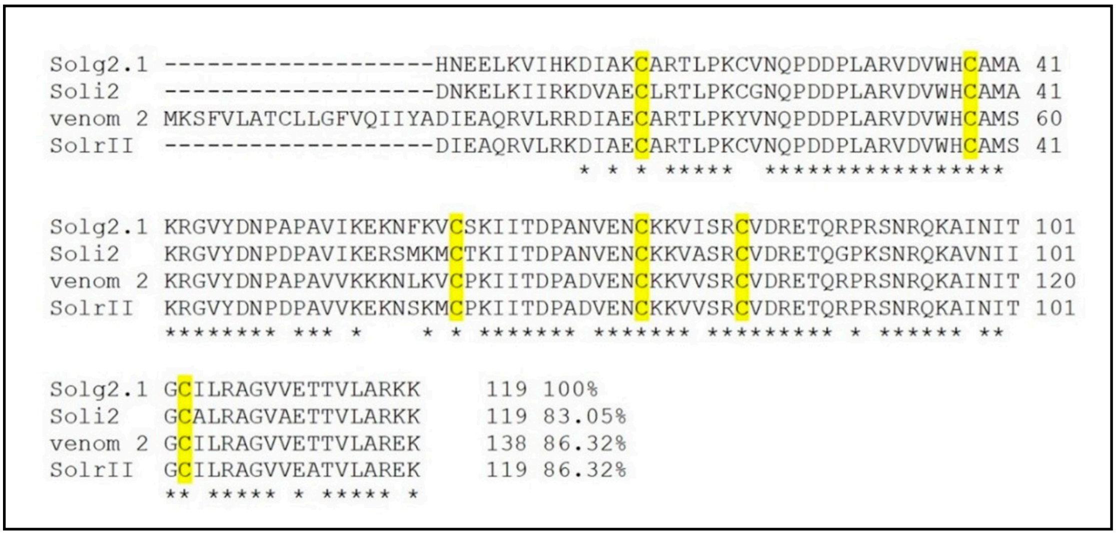

```
Solg2.1  --------------------HNEELKVIHKDIAKCARTLPKCVNQPDDPLARVDVWHCAMA 41
Soli2    --------------------DNKELKIIRKDVAECLRTLPKCGNQPDDPLARVDVWHCAMA 41
venom 2  MKSFVLATCLLGFVQIIYADIEAQRVLRRDIAECARTLPKYVNQPDDPLARVDVWHCAMS 60
SolrII   --------------------DIEAQRVLRKDIAECARTLPKCVNQPDDPLARVDVWHCAMS 41
                                       * * * *****  *****************

Solg2.1  KRGVYDNPAPAVIKEKNFKVCSKIITDPANVENCKKVISRCVDRETQRPRSNRQKAINIT 101
Soli2    KRGVYDNPDPAVIKERSMKMCTKIITDPANVENCKKVASRCVDRETQGPKSNRQKAVNII 101
venom 2  KRGVYDNPAPAVVKKKNLKVCPKIITDPADVENCKKVVSRCVDRETQRPRSNRQKAINIT 120
SolrII   KRGVYDNPDPAVVKEKNSKMCPKIITDPADVENCKKVVSRCVDRETQRPRSNRQKAINIT 101
         ******** *** *    * * ******* ******* ********* * ****** **

Solg2.1  GCILRAGVVETTVLARKK     119 100%
Soli2    GCALRAGVAETTVLARKK     119 83.05%
venom 2  GCILRAGVVETTVLAREK     138 86.32%
SolrII   GCILRAGVVEATVLAREK     119 86.32%
         ** ***** * ***** *
```

Figure S2. Multiple alignment of Sol g 2.1 amino acids sequence with other species of allergen venom based on NCBI databases was illustrated. The sequences included Sol i 2 (2YGU_A), allergen venom 2 (XP_025991263.1), and Sol r II (P35776.2): star (*) shows conserve sequence and yellow labeled shows cysteine residue.

Table S1. Shows number of crickets paralyzed after being treated with *S. geminata* crude venom

Crude venom (μg/g BW)	Number of paralysis crickets		
	Replicate 1	Replicate 2	Replicate 3
100	6	6	6
75	4	3	4
50	1	1	2
25	0	0	0

Table S2. Shows number of crickets paralyzed after being treated with various concentrations of piperidine alone and the piperidine plus rSol g 2.1 protein

**piperdine (%v/v)**	**Number of paralysis crickets**					
	Piperidine alone			Piperidine plus rSol g 2.1		
	Rep 1	Rep 2	Rep 3	Rep 1	Rep 2	Rep 3
0.3	6	6	6	6	6	6
0.2	6	6	6	6	6	5
0.1	4	3	4	5	4	4
0.05	2	2	1	3	3	2
0.025	1	0	0	1	1	1
0.012	0	0	0	0	1	0

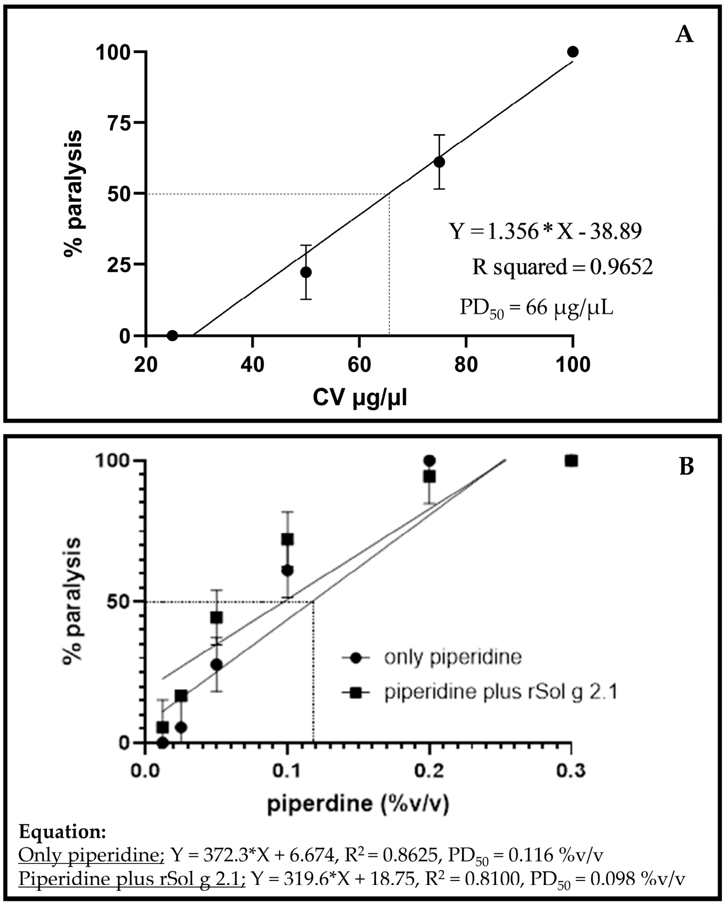

Figure S3. The percentage paralysis of the crickets after being injected with treatments were shown, including various concentrations of S. geminata crude venom (100, 75, 50, and 25 μg/g BW) (A), 2-methyl piperidine (0.3, 0.2, 0.1, 0.05, 0.025, and 0.012 % v/v) and the various concentrations of 2-methyl piperidine mixed with 0.85 μg/g BW of rSol g 2.1 (B). Each treatment was injected into six crickets, triplicate.

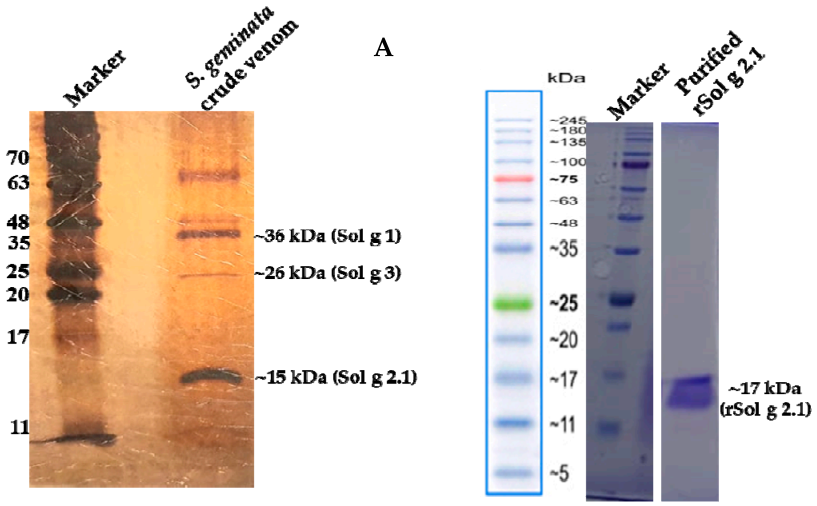

Figure S4. (A): An SDS-PAGE analysis using silver staining was conducted on *Solenopsis geminata* crude venom. The results revealed a prominent protein band at around 15 kDa, corresponding to the Sol g 2.1

protein, which represents a significant component of the venom. (B): In another SDS-PAGE analysis, using Coomassie brilliant blue G-250 staining, the purified recombinant Sol g 2.1 protein (rSol g 2.1) demonstrated an expected molecular weight of 17 kDa.

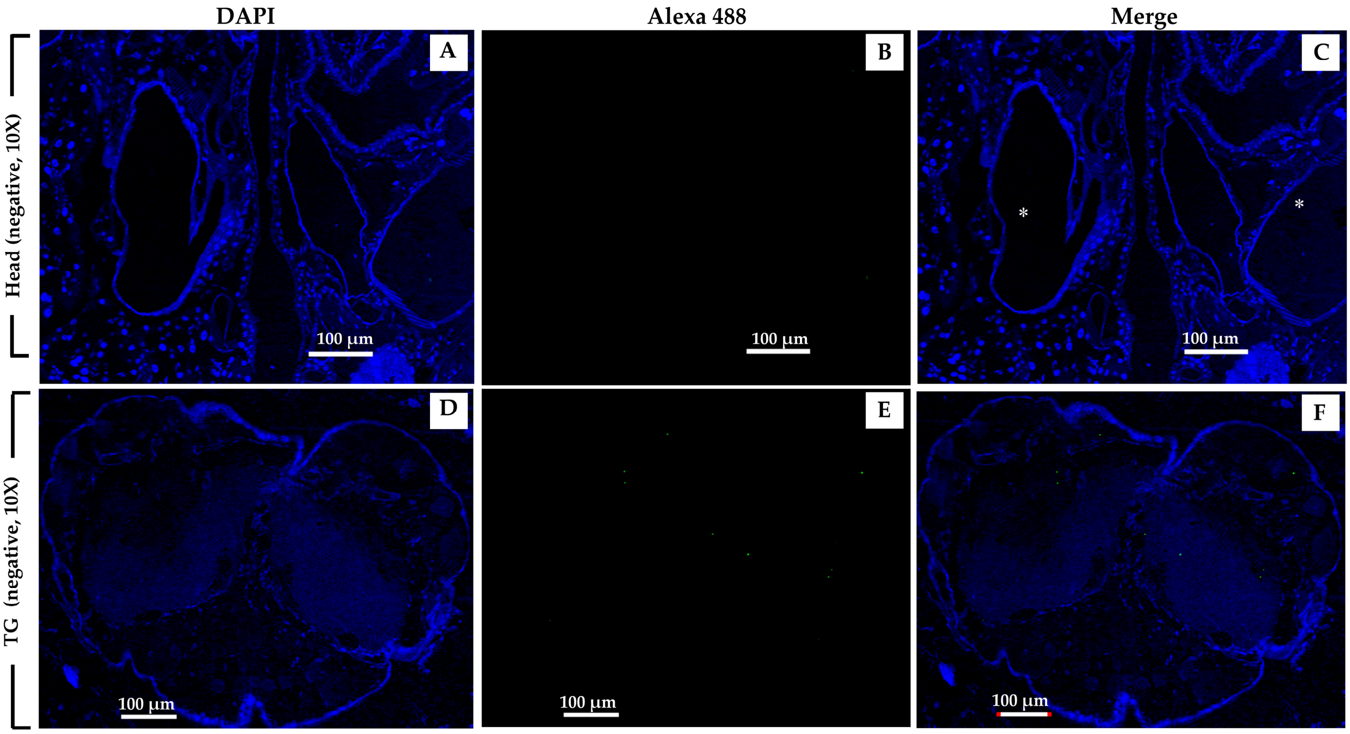

Figure S5. Negative control section of confocal microscopic photographs of the frontal section of the cricket's head (A-C) and the thoracic ganglion's longitudinal section (D-F), after incubating with pre-immune serum instead of anti-Sol g 2.1 primary antibody. (A-F): No immunoreactivity (green, Alexa 488) was observed in the negative control section. (A,C,D,F): The nuclei staining in the sections were shown in blue (DAPI).
